# Carnitine octanoyltransferase is important for the assimilation of exogenous acetyl-L-carnitine into acetyl-CoA in mammalian cells

**DOI:** 10.1016/j.jbc.2022.102848

**Published:** 2022-12-30

**Authors:** Jake Hsu, Nina Fatuzzo, Nielson Weng, Wojciech Michno, Wentao Dong, Maryline Kienle, Yuqin Dai, Anca Pasca, Monther Abu-Remaileh, Natalie Rasgon, Benedetta Bigio, Carla Nasca, Chaitan Khosla

**Affiliations:** 1 Department of Chemical Engineering, Stanford University, Stanford, California, USA; 2 Department of Chemistry, Stanford University, Stanford, California, USA; 3 Division of Neonatology, Department of Pediatrics, Stanford University, Stanford, California, USA; 4 Department of Genetics, Stanford University, Stanford, California, USA; 5 Sarafan ChEM-H, Stanford, California, USA; 6 Department of Psychiatry and Behavioral Sciences, Stanford University, Stanford, California, USA; 7 Department of Psychiatry, Grossman School of Medicine, New York University, New York, New York, USA; 8 Department of Neuroscience and Physiology, New York University Neuroscience Institute, Grossman School of Medicine, New York University, New York, New York, USA; 9 Harold and Margaret Milliken Hatch Laboratory of Neuroendocrinology, The Rockefeller University, New York, New York, USA

**Keywords:** acetyl coenzyme A (acetyl-CoA), cell metabolism, energy metabolism, metabolic regulation, enzyme turnover, mitochondrial metabolism, peroxisome, acetylcarnitine, BHB, β-hydroxybutyrate, CPT, carnitine palmitoyltransferase, CRAT, carnitine acetyltransferase, CROT, carnitine octanoyltransferase, DTNB, 5,5′-dithio-bis-(2-nitrobenzoic acid), LAC, acetyl-L-carnitine

## Abstract

In eukaryotes, carnitine is best known for its ability to shuttle esterified fatty acids across mitochondrial membranes for β-oxidation. It also returns to the cytoplasm, in the form of acetyl-L-carnitine (LAC), some of the resulting acetyl groups for posttranslational protein modification and lipid biosynthesis. While dietary LAC supplementation has been clinically investigated, its effects on cellular metabolism are not well understood. To explain how exogenous LAC influences mammalian cell metabolism, we synthesized isotope-labeled forms of LAC and its analogs. In cultures of glucose-limited U87MG glioma cells, exogenous LAC contributed more robustly to intracellular acetyl-CoA pools than did β-hydroxybutyrate, the predominant circulating ketone body in mammals. The fact that most LAC-derived acetyl-CoA is cytosolic is evident from strong labeling of fatty acids in U87MG cells by exogenous ^13^C_2_-acetyl-L-carnitine. We found that the addition of *d*_3_-acetyl-L-carnitine increases the supply of acetyl-CoA for cytosolic posttranslational modifications due to its strong kinetic isotope effect on acetyl-CoA carboxylase, the first committed step in fatty acid biosynthesis. Surprisingly, whereas cytosolic carnitine acetyltransferase is believed to catalyze acetyl group transfer from LAC to coenzyme A, *CRAT*^−/−^ U87MG cells were unimpaired in their ability to assimilate exogenous LAC into acetyl-CoA. We identified carnitine octanoyltransferase as the key enzyme in this process, implicating a role for peroxisomes in efficient LAC utilization. Our work has opened the door to further biochemical investigations of a new pathway for supplying acetyl-CoA to certain glucose-starved cells.

In eukaryotes, the zwitterionic metabolite carnitine serves a critical role in energy homeostasis by transporting esterified fatty acids across the inner mitochondrial membrane for β-oxidation (1, 2, 3, 4, 5, 6). At the same time, it also returns some of the acetyl-CoA products of β-oxidation to the cytosol in the form of acetyl-L-carnitine (LAC) (3, 7). These CoA:carnitine transacylation reactions are catalyzed by the well-known enzymes carnitine acetyltransferase (CRAT) and carnitine palmitoyltransferase (CPT) (1, 4, 5, 6) (Fig. 1).

**Figure 1:**
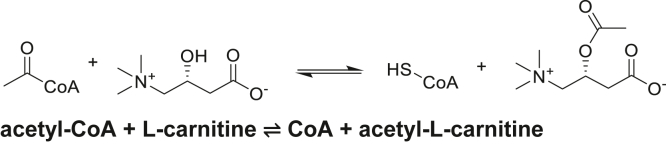
**Carnitine acetyltransferase reaction**.

Although low (micromolar) levels of both carnitine and LAC are found in human blood (8, 9), extracellular forms of these metabolites are generally not considered to have specific metabolic functions. It is unclear how exogenous LAC is metabolized, especially under glucose-limited growth conditions. A plausible hypothesis might involve uptake of LAC by the carnitine transporter SLC22A5/OCTN2 into certain glucose-starved cells, followed by CRAT-catalyzed transfer of its acetyl group into the cytosolic acetyl-CoA pool. In turn, an increase in acetyl-CoA flux could fuel cytosolic demands such as lipid synthesis as well as mitochondrial energetic needs. While qualitatively straightforward, the quantitative details of this hypothesis are problematic, given the relatively low LAC concentrations in the bloodstream even after dietary supplementation (10). A better definition of the pathway(s) by which exogenous LAC can alter cellular metabolism is therefore needed to increase our knowledge of energy homeostasis.

Here we use genetic and biochemical approaches to establish how exogenous LAC alters acetyl-CoA metabolism in U87MG, a malignant glioma cell line, especially under glucose limited conditions. We report that ^13^C_2_-acetyl-L-carnitine (^13^C_2_-LAC) is converted into ^13^C_2_-acetyl-CoA with overall efficiency that is highly dependent on glucose concentration. Both ATP generation and lipid biosynthesis are altered, highlighting mitochondrial and cytoplasmic effects. Using ^13^C_4_-β-hydroxybutyrate (^13^C_4_-BHB) as a comparator, we interrogated the relative importance of ^13^C_2_-LAC metabolism and ketone body metabolism in response to glucose restriction in different types of cultured mammalian cells. Perhaps most remarkably, a systematic analysis of homologous carnitine acyltransferases led to an unexpected finding regarding the role of peroxisomes in LAC metabolism.

## Results

### Analysis of LAC metabolism in U87MG cells

**Figure 2:**
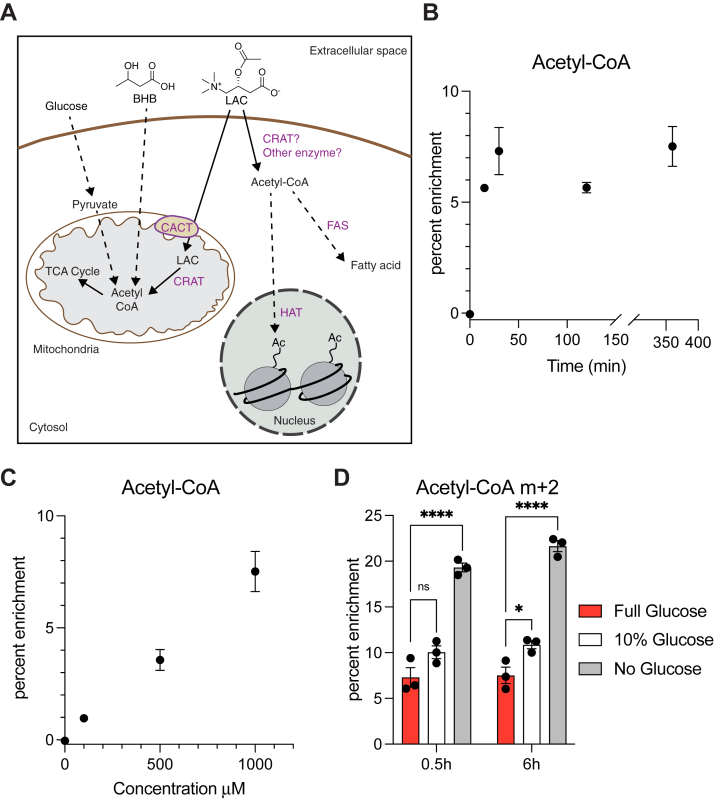
**LAC contributes to cytosolic acetyl-CoA pools in a concentration, time, and glucose-dependent manner in U87MG glioma cells.***A*, proposed utilization of exogenous acetyl-L-carnitine (LAC) in central metabolism. *B*, enrichment of M + 2 acetyl-CoA cultured with 1 mM unlabeled LAC and subsequently with 1 mM ^13^C_2_ LAC for specific time periods. *C*, fractional enrichment of m + 2 acetyl-CoA in U87 cells cultured for 6 h with specified concentrations of ^13^C_2_ LAC. *D*, fractional enrichment of m + 2 acetyl-CoA in U87 cells cultured with 1 mM ^13^C_2_ LAC at specified glucose concentrations and time points. Statistical significance was calculated by two-way ANOVA. Each point represents a biological replicate and error bars represent standard error of the mean. ∗*p* ≤ 0.05; ∗∗∗∗*p* ≤ 0.0001. BHB, β-hydroxybutyrate; CRAT, carnitine acetyltransferase.

Based on prior literature precedent, we anticipated that exogenous LAC is taken up into the cytosol by cells *via* the carnitine transporter SLA22A5/OCTN2 (11, 12), where it is converted into acetyl-CoA by a carnitine acyltransferase, most likely CRAT (Fig. 2*A*). Whole-cell metabolite extracts of U87MG cells were therefore analyzed by LC-MS after treatment with ^13^C_2_-LAC, *d*_3_-LAC, or vehicle control under normal culture conditions including 5 mM glucose (Fig. S1). Our measurements presumably reflected cytosolic acetyl-CoA concentrations given the limited volume of mitochondria in most cells and the comparable concentrations of acetyl-CoA in the cytosol *versus* mitochondria (13, 14). Formation of ^13^C_2_-acetyl-CoA was rapid (Fig. 2*B*) with steady-state conversion being achieved in less than 30 min following addition of ^13^C_2_-LAC (Fig. 2*B*). However, at physiological concentrations of exogenous LAC (∼10 μM) (9, 10), acetyl-CoA labeling was limited; a concentration of ∼1 mM was required to observe substantial enrichment under normal growth conditions (Fig. 2*C*). We therefore reduced glucose (to 0.5 mM corresponding to 10% of standard conditions, or no glucose) and observed a significant increase in acetyl-CoA labeling (Fig. 2*D*). Thus, acetyl-CoA generation is regulated by glucose availability, and LAC switches from a weak contributor to the cell’s central carbon metabolism to a more significant acetyl-CoA source under glucose limited conditions.

**Figure 3:**
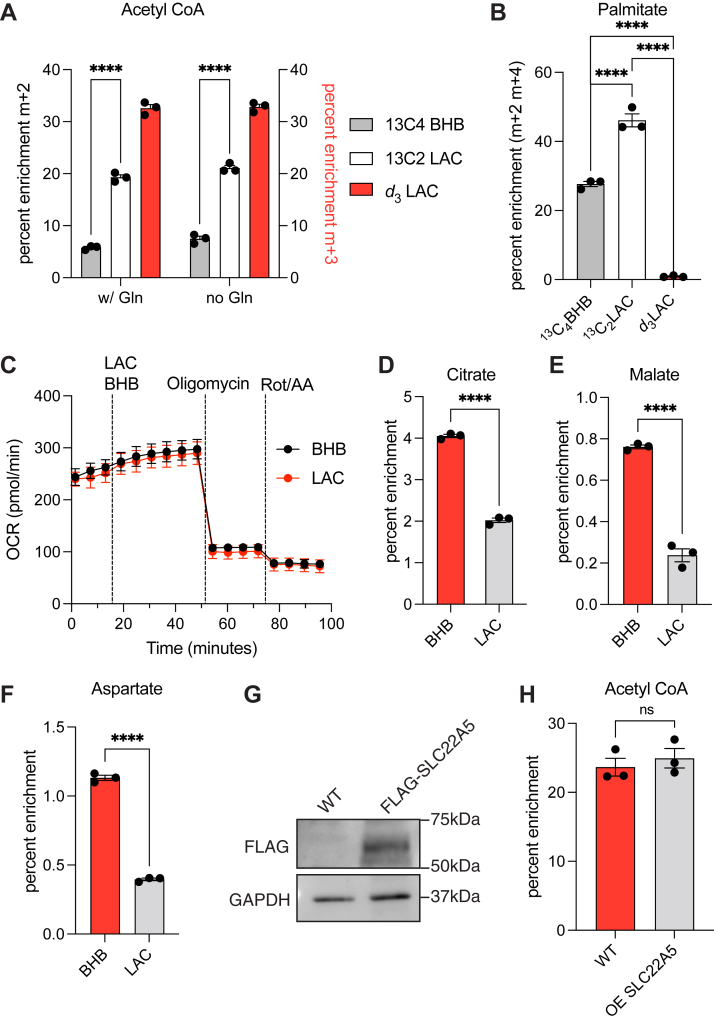
**Differential partitioning and utilization of exogenous LAC in U87MG glioma cells.***A*, fractional enrichment of whole-cell m + 2, m + 3 acetyl-CoA in cells cultured without glucose for 6 h in the presence of 1 mM of ^13^C_4_ BHB or ^13^C_2_ LAC or *d*_3_-LAC, with or without 4 mM Glutamine (Gln). Statistical significance between BHB and ^13^C_2_ LAC labeling was calculated by multiple *t*-tests. *B*, fractional enrichment of palmitic acid (sum m + 2 m + 4) in U87MG cells cultured with 0.5 mM glucose for 48 h with 1 mM of specified metabolite. Statistical significance was calculated by one-way ANOVA. *C*, cellular oxygen consumption rates from glycolysis and mitochondrial respiration upon treatment with BHB or LAC measured using Seahorse analyzer under glucose starvation. *D*–*F*, fractional enrichment of tricarboxylic acid intermediates in cells cultured with 0.5 mM ^13^C_4_ BHB or 1 mM ^13^C_2_ LAC in the absence of glucose for 6h. Statistical significance was calculated by unpaired *t* tests. *G*, immunoblot comparing expression of FLAG-tagged SLC22A5 in U87 after transient transfection for overexpression. *H*, fractional enrichment of m + 2 acetyl-CoA in U87 cells overexpressing SLC22A5 cultured without glucose with 1 mM ^13^C_2_ LAC for 6 h. Statistical significance was calculated by unpaired *t* test. Each point represents a biological replicate and error bars represent standard error of the mean. ∗∗∗∗*p* ≤ 0.0001. BHB, β-hydroxybutyrate; LAC, acetyl-L-carnitine.

To gain a better sense of the capacity of LAC to sustain central carbon metabolism in the absence of glucose, we sought to compare it to β-hydroxybutyrate (BHB), a canonical ketone body that is synthesized by the liver in response to glucose starvation and transported *via* systemic circulation as an acetyl-CoA source (15). Under equivalent conditions, ^13^C_2_-LAC labeled whole-cell acetyl-CoA considerably more effectively than ^13^C_4_-BHB (Fig. 3*A*). The presence of glutamine in the media did not significantly alter this finding, suggesting that anaplerosis is limited under these conditions (Fig. 3*A*). Given that cytosolic acetyl-CoA pools comprise a dominant portion of whole-cell acetyl-CoA pools and that cytosolic acetyl-CoA is primarily used for fatty acid biosynthesis in growing cells, ^13^C_2_-LAC also labeled palmitic acid in U87MG cells more effectively than ^13^C_4_-BHB (Fig. 3*B*). Furthermore, whereas *d*_3_-LAC is a much poorer source of acetyl-CoA for lipogenesis than ^13^C_2_-LAC (Fig. 3*B*) due to kinetic isotope effects of acetyl-CoA carboxylase, the first committed step in fatty acid biosynthesis (16), addition of *d*_3_-LAC to the culture medium results in more robust labeling of whole-cell acetyl-CoA than does ^13^C_2_-LAC (Fig. 3*A*).

To compare the ability of LAC and BHB to fulfill mitochondrial acetyl-CoA demand in the absence of glucose-derived pyruvate, the oxygen consumption of U87MG cells starved of glucose was monitored. In the presence of either carbon source, cells consume oxygen at a similar rate (Fig. 3*C*). Taken together, the above data imply that (i) unlike ketone bodies which are predominantly metabolized in the mitochondria, LAC contributes to acetyl-CoA metabolism in both the cytosol (where this central carbon intermediate primarily undergoes anabolic transformations) and the mitochondria (where it is primarily catabolized) and (ii) notwithstanding this difference, the oxidative flux of acetyl-CoA through the Krebs cycle is comparable in both cases. A key corollary of these findings is that the Krebs cycle is more active in the presence of BHB than LAC. In the presence of BHB, the Krebs cycle must export carbon in the form of citrate and oxaloacetate to the cytosol to fuel anabolic processes such as lipid synthesis and aspartate synthesis, respectively; these partial reactions of the Krebs cycle would not contribute NADH to fuel increased oxygen consumption. To verify greater Krebs cycle activity in response to BHB, mass spectrometric analysis was undertaken of Krebs cycle intermediates in the presence of ^13^C_2_-LAC or ^13^C_4_-BHB. As expected, malate, citrate, and aspartate (an oxaloacetate surrogate) exhibited higher m + 2 enrichment in the presence of ^13^C_4_-BHB (Fig. 3, *D*–*F*).

Finally, to establish whether LAC transport into U87MG cells limited its ability to contribute to intracellular acetyl-CoA pools, the carnitine importer SLC22A5/OCTN2, which is also believed to transport LAC (12), was overexpressed in this glioma cell line (Fig. 3*G*). No difference was observed in acetyl-CoA labeling between wildtype and SLC22A5-overexpressing cells, suggesting that the rate of cytosolic uptake does not limit acetyl-CoA generation from LAC (Fig. 3*H*).

### Unraveling the pathway for LAC metabolism in U87MG cells

**Figure 4:**
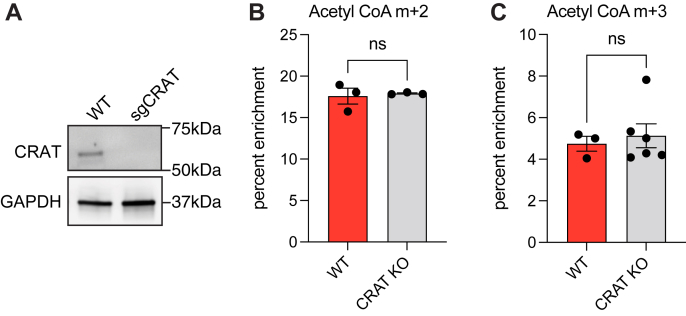
**Lack of CRAT requirement for LAC conversion to acetyl-CoA.***A*, immunoblot comparing CRAT expression in U87 cells edited with CRISPR/Cas9 to target CRAT. *B*, fractional enrichment of m + 2 acetyl-CoA in wildtype and CRAT-edited cells cultured with 1 mM ^13^C_2_ LAC, no glucose. Statistical significance was calculated by unpaired *t* test. *C*, fractional enrichment of m + 3 acetyl-CoA in wildtype and CRAT-edited cells cultured in standard glucose conditions and 1 mM *d*_3_-LAC. Statistical significance was calculated by unpaired *t* test. Each point represents a biological replicate and error bars represent standard error of the mean. CRAT, carnitine acetyltransferase; LAC, acetyl-L-carnitine.

**Figure 5:**
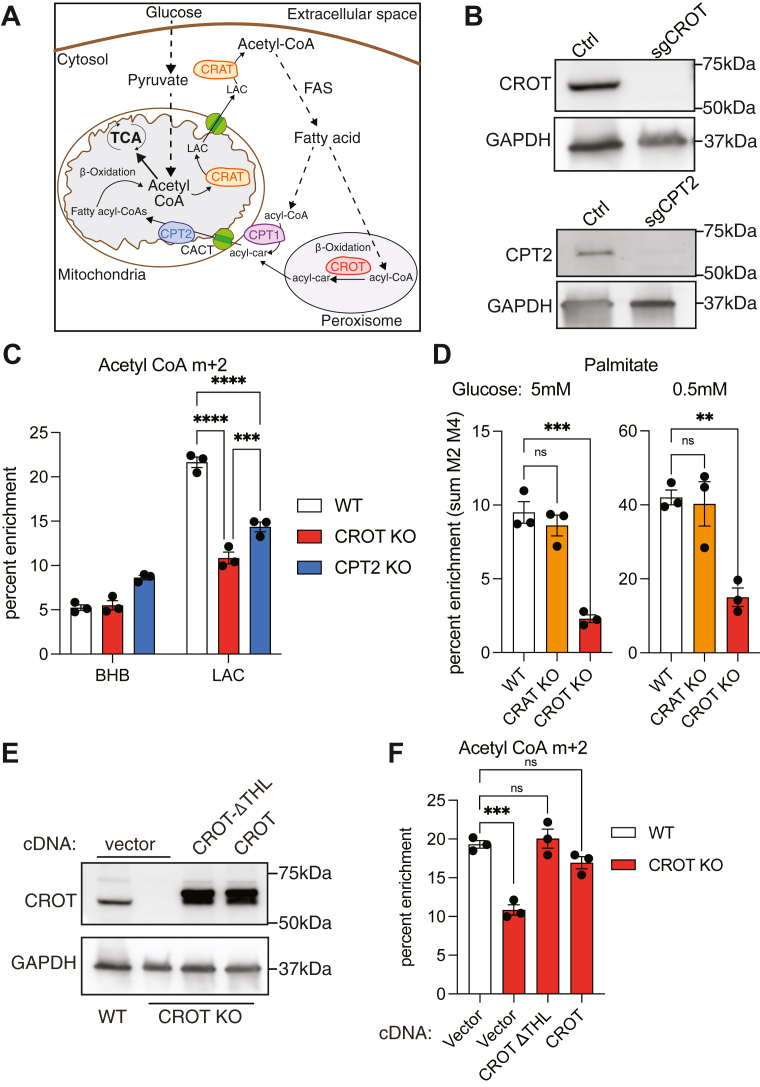
**CROT contributes to acetyl-CoA generation from acetyl-carnitine.***A*, cellular distribution of carnitine acyltransferases in facilitating fatty acid metabolism. *B*, immunoblot comparing expression of CROT/CPT2 in which Cas9 editing was used to target a safe-targeting region or CROT/CPT2. *C*, fractional enrichment of M + 2 acetyl-CoA in control and CROT/CPT2-knockout cells cultured in the absence of glucose and the addition of 1 mM ^13^C_4_ BHB or ^13^C_2_ LAC for 6 h. Statistical significance was calculated by two-way ANOVA. *D*, fractional enrichment of palmitic acid (sum m + 2 m + 4) in control and CRAT/CROT knockout cells cultured with 1 mM ^13^C_2_ LAC and specified glucose concentrations for 24 h. Statistical significance was calculated by one-way ANOVA. *E*, immunoblot comparing CROT expression in wildtype, CROT-null, and CROT cDNA-rescued cells. ΔTHL indicates truncation of the C-terminal peroxisomal targeting sequence. *F*, fractional enrichment of m + 2 acetyl CoA after cDNA rescue in U87MG cells cultured with ^13^C_2_ LAC without glucose for 6 h. Statistical significance was calculated by one-way ANOVA. Each point represents a biological replicate and error bars represent standard error of the mean. ∗∗*p* ≤ 0.01; ∗∗∗*p* ≤ 0.001; ∗∗∗∗*p* ≤ 0.0001. CPT, carnitine palmitoyltransferase; CRAT, carnitine acetyltransferase; CROT, carnitine octanoyltransferase; LAC, acetyl-L-carnitine.

In mammals, CRAT would reasonably be expected to convert LAC into acetyl-CoA due to its presence in the cytosol (17, 18) and its preference for short-chain acyl donors (17). To verify this prediction, CRAT-knockout U87MG cells were validated by Western blotting (Fig. 4*A*) and subsequently treated with ^13^C_2_-LAC. To our surprise, no changes were observed in the labeling intensity of acetyl-CoA, suggesting that the knockout cell line converted labeled LAC to acetyl-CoA with similar efficiency compared to wildtype cells (Fig. 4, *B* and *C*). As elaborated below, further validation of this unexpected finding also emerged from palmitic acid profiling. It prompted us to hypothesize that one or more alternative enzymes were able to catalyze acetyl transfer in the absence of CRAT. Specifically, the family of carnitine acyltransferases includes the medium-chain octanoyltransferase (CROT) and the long-chain palmitoyltransferase (CPT2) in addition to CRAT (Fig. 5*A*). Whereas CPT2 is localized on the inner mitochondrial matrix, CROT is predominantly a peroxisomal enzyme (19).

Individual gene knockouts of CROT and CPT2 were constructed in U87MG cells (Fig. 5*B*). Remarkably, CROT-knockout cells exhibited significant reduction in whole-cell acetyl-CoA and palmitate labeling by ^13^C_2_-LAC (Fig. 5, *C* and *D*), highlighting an important role for peroxisomal CROT in acetyl transfer. As predicted from the data in Figure 4, robust labeling of palmitic acid was retained in CRAT-knockout cells under equivalent conditions (Fig. 5*D*). CPT2-knockout cells also showed a reduced ability to label acetyl-CoA and palmitate under equivalent conditions, but the decrease was not as pronounced as that observed in the CROT-knockout cell line. Previous studies have shown that CPT2 disfavors short-chain substates (20, 21). To assess the role of CPT1 in LAC metabolism, etomoxir, an irreversible inhibitor of CPT1 that also inhibits CROT (22), was also tested and shown to reduce palmitate labeling (Fig. S2). Thus, even though CRAT is well-expressed in U87MG cells, LAC metabolism requires the activity of other carnitine acyltransferases, especially one localized in the peroxisome. This unexpected property of U87MG cells could either be regulated at the level of enzyme activity or *via* availability of CoASH in different subcellular compartments (Fig. 5*A*).

To verify the role of CROT in LAC metabolism, CROT^−/−^ cells were transfected with a cDNA encoding the wildtype CROT gene in the presence or absence of its (C terminal) peroxisome targeting sequence (Fig. 5*E*). Both cell lines rescued LAC-mediated acetyl-CoA labeling to wildtype levels, validating CROT as a key enzyme in acetyl-CoA production from exogenous LAC under glucose-limited conditions (Fig. 5*F*). The resulting peroxisomal acetyl-CoA is expected to be exported into the cytosol *via* peroxisomal transporters SLC25A17 and possibly ABCD1-3 (23, 24).

### Comparative biochemical analysis of CRAT and CROT

To gain insights into differences between CRAT and CROT that enable preferential utilization of the latter enzyme for the metabolism of exogenous LAC, we undertook comparative biochemical analysis of these two homologous enzymes (25). Recombinant human CRAT and CROT were expressed in and purified from *E. coli* and subjected to steady-state kinetic analysis in the presence of a set of acyl-CoA substrates (Table 1 and Fig. S3). Both CRAT and CROT have relatively broad specificity (*k*_*cat*_/*K*_*M*_) for short-to-medium length acyl chains, although CROT appears to have evolved some ability to discriminate against acetyl-CoA relative to C_3_ to C_8_ substrates. While CRAT exhibited a generally higher turnover rate (*k*_*cat*_) than CROT under our specified *in vitro* assay conditions, it must be borne in mind that CROT operates predominantly in the peroxisome in mammalian cells and may therefore have evolved different optimal turnover characteristics. In aggregate, our biochemical data support the premise that CROT can transacylate acetyl groups from LAC to coenzyme A, so long as nonlimiting (50–100 μM) concentrations of the latter substrate are available in the peroxisome. In this context, it is noteworthy that the cytosol can be often starved of CoASH with concentrations dropping as low as 20 μM (26). Perhaps CoASH availability in the cytosol represents a control mechanism to channel LAC that has been taken up to peroxisomal CROT.

**Table 1:** *K*_*M*_, *k*_*cat*_, *k*_*cat*_/*K*_*M*_, and *k*_*rel*_ values for CRAT and CROT with various acyl-CoA substrates

Acyl-CoA	CROT				CRAT			
	*K*_*M*_ (μM)	*k*_*cat*_ (s^−1^)	*k*_*cat*_/*K*_*M*_ (μM^−1^ s^−1^)	*k*_*rel*_	*K*_*M*_ (μM)	*k*_*cat*_ (s^−1^)	*k*_*cat*_/*K*_*M*_ (μM^−1^ s^−1^)	*k*_*rel*_
Acetyl-CoA	77	1.4	0.02	1.0	25	49	2.0	1.0
Propionyl-CoA	49	8.4	0.17	9.4	50	77	1.5	0.75
Butyryl-CoA	41	10	0.24	13	58	66	1.1	0.55
Hexanoyl-CoA	60	18	0.30	17	55	47	0.9	0.44
Octanoyl-CoA	24	13	0.54	30	21	35	1.7	0.85

k_rel_ calculated by dividing k_cat_/K_M_ (acyl-CoA, CRAT or CROT) by k_cat_/K_M_ (acetyl-CoA, CRAT or CROT).

### Utilization of LAC by other cell lines

**Figure 6:**
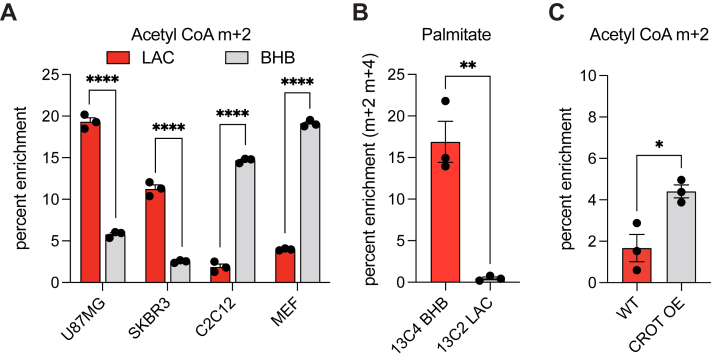
**Acetyl-carnitine utilization is cell-type specific**. *A*, fractional enrichment of whole-cell m + 2 acetyl-CoA in cells cultured with no glucose for 6 h in the presence of 1 mM of ^13^C_4_ BHB or ^13^C_2_ LAC. Statistical significance was calculated by multiple *t* tests. *B*, palmitic acid (sum m + 2 m + 4) enrichment in C2C12 myoblasts cultured for 48 h in glucose-containing medium with 1 mM of the appropriate metabolite. Statistical significance was calculated by unpaired *t* test. *C*, acetyl-CoA m + 2 enrichment in C2C12 myoblasts, overexpressing CROT or wildtype, upon culture with 1 mM ^13^C_2_ LAC for 30 min in media without glucose. Statistical significance was calculated by unpaired *t* test. Each point represents a biological replicate and error bars represent standard error of the mean. ∗*p* ≤ 0.05; ∗∗*p* ≤ 0.01; ∗∗∗∗*p* ≤ 0.0001. BHB, β-hydroxybutyrate; CROT, carnitine octanoyltransferase; LAC, acetyl-L-carnitine.

To investigate the generality of the above findings among other cell types, we compared selected cancerous and noncancerous cell lines using some of the assays described above. Whereas SK-BR-3, a breast adenocarcinoma cell line, displayed similar characteristics to U87MG (Fig. 6*A*), the noncancerous C2C12 myoblast and mouse embryonic fibroblast lines exhibited the opposite trend in that BHB outperformed LAC in labeling acetyl-CoA (Fig. 6*A*) and palmitate (Fig. 6*B*). Our data suggest that while many noncancerous cell types are more reliant on standard ketone body metabolism to meet their energetic needs under glucose-limited conditions, cancer cells have upregulated the LAC utilization pathway highlighted in this report.

To test whether CROT overexpression is sufficient to switch a cell’s preference between BHB and LAC, human CROT was overexpressed in C2C12 myoblasts (Fig. S4). The resulting cell line exhibited a modest increase in acetyl-CoA labeling, but not at levels observed in U87MG and SKBR3 (Fig. 6*C*), implying that additional factors are required to enhance a cell’s ability to efficiently utilize LAC as an anabolic carbon and energy source.

## Discussion

The role of LAC in the context of the carnitine shuttle has been extensively studied (1, 2, 3, 4, 7). Here, we characterize the cellular metabolism of exogenous LAC in the context of glucose limitation. Under these conditions, some but not all cells utilize LAC to produce acetyl-CoA with considerably higher efficiency than BHB, a canonical ketone body. Surprisingly, conversion of LAC into acetyl-CoA in these cells does not appear to primarily utilize the more straightforward pathway involving the action of cytosolic CRAT. Instead, LAC is routed into the peroxisome, where it is converted into acetyl-CoA by a homologous peroxisomal enzyme, CROT. While the biochemical logic of this multicompartmental pathway for exogenous LAC metabolism remains to be elucidated, a plausible explanation for its surprising efficiency lies in CoASH availability. Whereas cytosolic CoASH concentrations are low under at least some conditions (14, 26), CoASH in the peroxisome can be expected to be abundant due to its specialization as an organelle dedicated to β-oxidation of fatty acids.

Although it may be tempting to consider LAC as a ketone body capable of supplying acetyl-CoA to a cell under glucose-restricted conditions, notable metabolic differences are apparent when one compares LAC to BHB. Specifically, cells that utilize LAC exhibit greater anabolic activity in the cytosol without compromising oxidative phosphorylation activity in the mitochondria.

Overall, this work provides evidence that LAC metabolism is a key pathway for carbon and energy metabolism of glucose-starved cells. These data lay the foundation for functional studies in rodent models as well as engineering efforts to derivatize LAC for improved pharmacological properties.

## Experimental procedures

### Chemicals

**Table:** 

Acetyl-L-carnitine hydrochloride ^13^C_2_	This study	
Acetyl-L-carnitine hydrochloride *d*_3_	This study	
Acetyl-L-carnitine hydrochloride	J&K Scientific	51695-4
L(-)-carnitine hydrochloride	Arcos Organics	230280050
Sodium D-3-hydroxybutyrate ^13^C_4_	Cambridge Isotope Laboratories, Inc	CLM-3853
Trichloroacetic acid	Fisher Chemical	A322-100
Ellman’s reagent	G Biosciences	BC87
Acetonitrile HPLC Plus	Sigma Aldrich	34998
InfinityLab Deactivator Additive	Agilent Technologies	5191-4506
Ammonium acetate (Optima)	Fisher Chemical	A114-50
Hexanes, Optima	Fisher Chemical	H303-1
Methanol HPLC-Grade	Fisher Chemical	A452-1
Acetyl chloride	Thermo Scientific	AC219470250
RIPA buffer	Research Products International	R26200

### Cell culture

All cell lines were obtained from ATCC. U87MG cells were cultured in Dulbecco's modified Eagle's medium (DMEM) media, 1 g/l glucose (Corning #10-014-CV) supplemented with 10% FBS (Gemini #100-106), and 1% penicillin/streptomycin (Gibco #15140122). SK-BR-3 cells were cultured in McCoy’s 5A Medium (Gibco #16600082) supplemented with 10% FBS and 1% penicillin/streptomycin. C2C12 myoblasts and mouse embryonic fibroblasts (MEFs) were cultured in DMEM media, 1 g/l glucose supplemented with 10% FBS, and 1% penicillin/streptomycin.

Metabolite addition and inhibitor treatment experiments were performed in the media conditions listed with supplementation of the specified metabolite with equal volumes of vehicle control unless otherwise specified. Carnitine analogs and BHB were solubilized in water, and pH was adjusted to 7.5 with sterile-filtered NaOH as needed.

### Generation of CRISPR-edited cell lines

LentiCRISPR v2 (27) (Addgene) was used as the second-generation lentiviral backbone for all knockout lines. Sequences for all sgRNAs used in this study are listed in Table S1. The guide sequences were cloned into the lentiviral backbone using the CRISPick tool (28, 29). Lentiviral packaging plasmids (psPAX2, pMD2.G) were from Addgene. The lentiviral backbone plasmid (1 μg) containing the guide sequence and 500 ng of each of the packaging plasmids were co-transfected into HEK293T cells using Lipofectamine 3000 transfection reagent (Thermo Scientific). The viral media were exchanged after 24 h, harvested after 48 h, and passed through a 0.45 μm filter. U87MG cell lines were reverse-transduced by trypsinizing adherent cells and adding cell suspensions to viral media supplemented with 10 μg/ml polybrene. All lines were placed under relevant antibiotic selection beginning 72 h posttransduction and lasting until control (untransduced) cells completely died. Infected cells were selected for gene insertion using puromycin (2 μg/ml) or blasticidin (10 μg/ml).

### Cell line transfection/transduction for overexpression of target proteins

pcDNA3.1 encoding SLC22A5 was from Origene. Transient transfection was performed using Lipofectamine 3000 transfection reagent (Thermo Scientific). Cells were incubated overnight with lipofectamine and DNA, and media were replaced with fresh media. Forty-eight hours after transfection, cells were treated for labeling studies or harvested for Western blotting.

A synthetic gene encoding CROT (Twist Biosciences) was cloned into pLenti-EF1a using Gibson assembly. A variant without the C-terminal peroxisomal targeting sequence was cloned simultaneously (CROT ΔTHL). Lentiviral particles were packaged as described above for CRISPR knockouts, and transduction was performed as described earlier.

### Metabolite labeling assays

For LAC and BHB labeling assays, media were aspirated, and fresh medium containing specified concentrations of glucose/glutamine was added to cells along with ^13^C_2_ LAC or ^13^C_4_ BHB or *d*_3_-LAC. For studies comparing ^13^C_2_ LAC and ^13^C_4_ BHB labeling, equimolar concentrations of the two compounds were used despite the ability of BHB to generate two ^13^C_2_ acetyl-CoA equivalents. The use of equal concentrations was justified given our objective to measure flux in acetyl-CoA generation (as opposed to absolute yield) and the fact that the respective rate-limiting steps for conversion of LAC and BHB to acetyl-CoA are distinct.

### CoA metabolite extraction and LC-MS analysis

Metabolites were extracted using a modification of previously reported methods (30, 31). U87MG cells were removed from the incubator and placed on ice immediately. Media were aspirated, and 10% trichloroacetic acid was added immediately (800 μl to a 6 cm dish). Samples were placed at −80 °C for 30 min to maximize metabolic quenching. Cells were then thawed and scraped using a cell scraper, and the cell suspension was centrifuged at 16,000*g* for 15 min at 4 °C. The supernatant was purified using an Oasis HLB SPE column (Waters). The SPE eluate was dried under nitrogen and frozen at −80 °C until sample analysis.

LC-MS analysis was performed as follows. Dried samples were resuspended in 80% acetonitrile/20% 10 mM ammonium acetate, pH 9, then vortexed rapidly for 5 min, and centrifuged at 16,000*g* for 20 min at 4 °C. The supernatant was filtered and analyzed by LC-MS (Agilent 1290 HPLC coupled with Agilent 6545 Q-TOF AJS mass spectrometer). Samples were separated on an InfinityLab Poroshell 120 HILIC-Z column (2.1 × 150 mm) on a 1290 LC system. Buffers A: 10 mM ammonium acetate, pH 9, with 2.5 μM medronic acid; B: 85% acetonitrile/15% water containing 10 mM ammonium acetate, pH 9, and 2.5 μM medronic acid on a gradient from 90 to 50% B. Enrichment of isotopically labeled metabolites was determined using FluxFix (32) based on samples treated with no isotope tracer.

### Immunoblotting

Western Blotting for whole-cell extracts was performed as follows. U87MG cells were removed from culture plates using trypsin and washed twice with ice-cold PBS. Cells were lysed in RIPA buffer with HALT protease inhibitor by incubating on a rotating mixer at 4 °C for 20 min, and lysates were clarified by centrifugation at 16,000*g* at 4 °C for 20 min. Total protein content was quantified by Bradford before SDS-PAGE.

Cell extracts for CRAT western blotting were prepared as follows. Cells were harvested by trypsinization and resuspended in NKM buffer (1 mM Tris HCl, pH 7.4, 0.13 M NaCl, 5 mM KCl, and 7.5 mM MgCl_2_). Cells were pelleted and resuspended in homogenization buffer (10 mM Tris-HCl, pH 6.7, 10 mM KCl, 0.15 mM MgCl_2_, 1 mM PMSF, and 1 mM DTT) and homogenized in a Dounce homogenizer on ice for 30 strokes. The homogenized suspension was centrifuged, and the pellet was resuspended in 2xLaemmli sample buffer for gel electrophoresis.

Lysates were resolved by SDS–PAGE (Bio-Rad) at 180 V. Resolved proteins were transferred to nitrocellulose membranes (GE Healthcare). Membranes were blocked with 5% nonfat dry milk in TBST (Tris-buffered saline with Tween-20) for 1 h, then incubated overnight with primary antibodies in 5% nonfat dry milk in TBST at 4 °C. After incubation, membranes were washed three times with TBST for 5 min per wash and then incubated with the appropriate secondary antibodies diluted 1:10,000 in 5% nonfat dry milk for 1 h at room temperature. Membranes were then washed three times with TBST and visualized using ECL2 western blotting substrate (Thermo Scientific).

Antibodies used in this study were as follows: CRAT (Invitrogen #PA5-82800), CROT (Proteintech #13543-1-AP), FLAG-HRP (Biolegend #637312), CPT2 (Invitrogen #MA532314), GAPDH (Biolegend #649201), alpha Tubulin (Invitrogen #62204), HRP Donkey anti-rabbit IgG (minimal x-reactivity) (Biolegend #406401), and HRP Goat anti-mouse IgG (minimal x-reactivity) (Biolegend #405306).

### Fatty acid analysis

Fatty acids were derivatized into methyl esters following a published procedure (33). Methanolic HCl was prepared by addition of 200 μl acetyl chloride to 4 ml methanol. Media were aspirated from plates, and methanolic HCl (2 ml) was added to each well, and cells were removed by scraping. Cell suspensions were placed in glass tubes, and saponification was performed by incubation at 90 °C for 2 h. The saponified fatty acids were cooled to room temperature and extracted twice with hexanes (1 ml) by adding hexanes, vortexing, and transferring the top hexanes layer to a new vial. The samples were dried under nitrogen and resuspended in 200 μl hexanes for GC-MS analysis. GC-MS was performed on a HP 7890/5975 GC-MS on a DB-5 column with a 10:1 split ratio. Deuterium or carbon enrichment into palmitate was determined using FluxFix (32).

### Oxygen consumption rate assay

The oxygen consumption rate was measured using a Seahorse XF24 Extracellular Flux Analyzer (Agilent Technologies). U87MG cells and C2C12 cells were plated on tissue culture–treated XF24 24-well plates (Agilent Technologies) at 8 × 10^4^ cells per well in standard maintenance medium. The next day, the cells were washed twice with assay medium (Seahorse XF DMEM medium supplemented with 2 mM L-glutamine, no pyruvate or glucose) and changed to assay medium 30 min before the assay. Baseline measurements of oxygen consumption rate were obtained three times. 10 mM LAC/BHB was injected as the first drug treatment, followed by injections of 1.5 μM oligomycin and 0.5 μM rotenone/antimycin.

### Tricarboxylic acid metabolite extraction and LC-MS analysis

Cells were washed with ice-cold 0.9% saline solution on wet ice to remove extracellular metabolites. Cell plates were moved to dry ice and extraction solvent, 80% methanol (HPLC-grade)/20% water (HPLC-grade) with 500 nM isotopically labeled amino acids, was added to wells, and cells were removed with cell scrapers on dry ice. Samples were vortexed rapidly for 15 min at 4 °C, followed by centrifugation at 16,000*g* for 15 min at 4 °C. Supernatants were collected in new tubes and vortexed briefly before an additional centrifugation at 16,000*g* for 15 min at 4 °C. Supernatants were transferred to mass spectrometry vials for analysis.

LC-MS analysis was performed by a Vanquish LC system coupled with an ID-X Tribrid mass spectrometry (Thermo Scientific). An electrospray ionization probe was used for the ID-X. For the LC system, a SeQuant ZIC-pHILIC 150 × 2.1 mm column (Millipore Sigma 1504600001) preceded by a 20 × 2.1 mm (Millipore Sigma 1504380001) guard column was used to perform hydrophilic interaction chromatography. The mobile phases are 100% acetonitrile for B and 100% water with 20 mM ammonium carbonate and 0.1% ammonium hydroxide for A. The chromatographic gradient is as follows: initial, 80% B; 0 to 20 min, linear decrease from 80 to 20% B; 20 to 20.5 min, linear increase from 20 to 80% B; 20.5 to 29.5 min, hold at 80% B. Mobile phase flowrate, 0.15 mM/min; injection volume, 2.5 μl; autosampler temperature, 4 °C; column chamber temperature, ambient.

For the IDX MS system, the following parameters were used: orbitrap resolution, 120,000 AU; positive ionization voltage, 3 kV; negative ionization voltage, 2.5 kV; vaporizer temperature, 350 °C; ion transfer tube temperature, 275 °C; AGC target, 1 × 10^6^ AU; RF lens, 40%; maximum injection time, 80 ms; sheath gas, 40 AU; aux gas, 15 AU; full scan range, 70 to 1000 *m/z*; EASYIC internal calibration status, enabled.

For isotope enrichment assessment, Compound Discoverer 3.1 (Thermo Scientific) was used for stable isotope tracing analysis that automatically performs natural abundance corrections. A compound mass-list with validated retention time and tandem mass spectrum was used for metabolite identification. For detailed method, see https://doi.org/10.1038/s41586-022-05221-y.

### Recombinant protein expression and purification

Synthetic genes for *CRAT* and *CROT* (Twist Biosciences) were cloned into pET21 for protein expression. *E. coli* BL21(DE3) cells were transformed and cultured in LB media, supplemented with appropriate antibiotic. Cultures (1 l) were inoculated with overnight seed culture at v/v = 200:1, shaken at 37 °C and 250 rpm until an *A*_600_ of 0.7 was attained, then cooled to 20 °C, and induced with 200 μM isopropyl-β-D-thiogalactopyranoside (Gold Biotechnology). Cells were harvested after 16 h by centrifugation at 5000*g* for 7 min and resuspending in lysis buffer (50 mM phosphate, pH 8.0, 10 mM imidazole, 500 mM NaCl, 10% (v/v) glycerol). Cells were then lysed by sonication, and the lysate was clarified by centrifugation (27,000*g*, 60 min). The supernatant was incubated with 4 ml of Ni-NTA resin (Invitrogen, 50% slurry, pre-equilibrated with lysis buffer) for 1 h at 4 °C. The resin was washed with five column volumes of wash buffer (50 mM phosphate, pH 8.0, 25 mM imidazole, 300 mM NaCl, 10% (v/v) glycerol) and eluted with three column volumes elution buffer (50 mM phosphate, pH 8.0, 500 mM imidazole, 10% (v/v) glycerol). The eluant was applied to a 5 ml HiTrapQ HP column or a HiTrap SP column (GE Healthcare) depending on the predicted pI of each protein.

For anion exchange chromatography, columns were washed with 4CV of FPLC Buffer A (50 mM phosphate, pH 8.8, 10% (v/v) glycerol). Proteins were eluted with a gradient 0 to 100% FPLC Buffer B (50 mM phosphate, pH 8.2, 1 M NaCl, 10% (v/v) glycerol) over 20 column volumes. For cation exchange, columns were wash with 4CV of FPLC Buffer A (50 mM phosphate, pH 6.8, 10% (v/v) glycerol). Proteins were eluted with a gradient 0 to 100% FPLC Buffer B (50 mM phosphate, pH 6.8, 1 M NaCl, 10% (v/v) glycerol) over 20 column volumes. The eluted fractions were pooled and concentrated in an Amicon filter (Millipore) and flash frozen with 10% glycerol.

### Enzyme assays

A DTNB stock solution was prepared containing 25 mM sodium acetate and 5 mM DTNB in water. In addition, a 1 M Tris buffer at pH 7.8 was made. A master mix was created containing enzyme (CRAT or CROT), carnitine (or carnitine derivative), and 1 M Tris diluted to 0.1 M. 5 mM DTNB stock solution was added to a final concentration of 0.5 mM, and the absorbance at 405 nm was read for 3 min to allow the DTNB to fully react with any available cysteines on the enzyme. After 3 min, acyl-CoA was added to initiate the reaction, and the absorbance at 405 nm was read. To identify the extinction coefficient of the TNB ion (calculated to be 9148 M^−1^ cm^−1^), a calibration curve was obtained in 0.1 M Tris buffer (pH 7.8). Again, 5 mM DTNB stock solution was added to a final concentration of 0.5 mM, and the absorbance at 405 nm was read for 3 min. Finally, CoA at varying concentrations was added to initiate the reaction, and the absorbance at 405 nm was read for approximately 5 min. All assays were run in a half-area 96-well plate with a standard path length of 0.5 cm for 80 μl volume. This standard path length was used to correct the absorbance in all assays. Each of the Michaelis–Menten curves shown is the result of technical duplicates.

To calculate the concentration of each acyl-CoA species, the amount of total CoA (including acyl-CoA and free CoA) was first calculated using the absorbance at 260 nm. A calibration curve of CoA absorbance at 260 nm was established. Using this calibration curve, the total amount of CoA could be calculated. Next, the amount of hydrolyzed acyl-CoA was calculated by reacting the acyl-CoA with DTNB and reading absorbance at 405 nm. This absorbance was fit to the standard curve for CoA reacting with DTNB, and the same procedure to make the standard curve was followed. Finally, the amount of hydrolyzed acyl-CoA was subtracted from the total amount of CoA to give the amount of acyl-CoA added to each reaction.

### Synthesis of ^13^C_2_ acetyl-L-carnitine

To a stirred solution of L-carnitine (0.15 g, 1 eq) in ^13^C_2_-acetic acid (0.16 ml, 3 eq) was slowly added ^13^C_2_-acetyl chloride (0.12 ml, 1.8 eq), maintaining a temperature <25 °C. The mixture was then allowed to stir at room temperature for 3 h during which a white solid started to precipitate. Acetone and hexanes (4 ml) were added, and the suspension stirred at room temperature for 10 min to precipitate the product. The reaction mixture was filtered, washed with acetone (5 ml), and dried under vacuum overnight to give ^13^C_2_ acetyl-L-carnitine hydrochloride (0.15 g, 79%) as a white solid.

^1^H NMR (400 MHz, D_2_O) δ 5.65 (s, 1H), 3.91 (dd, *J* = 14.6, 8.8 Hz, 1H), 3.68 (d, *J* = 14.6 Hz, 1H), 3.21 (s, 9H), 2.90 to 2.72 (br m, 2H), 2.16 (dd, *J* = 130.7, 6.9 Hz, 3H).

### Synthesis of d_3_-acetyl-L-carnitine

To a stirred solution of L-carnitine (5.0 g, 31.0 mmol, 1 eq) in tetradeuteroacetic acid (5.68 ml, 93.1 mmol, 3 eq) was slowly added acetyl chloride-*d*_3_ (3.30 ml, 46.5 mmol, 1.5 eq), maintaining a temperature <35 °C. The gummy mixture was then allowed to stir at room temperature for 2 h during which a white solid started to precipitate. Acetone (40 ml) was added, and the suspension stirred at room temperature for 10 min. The reaction mixture was filtered, washed with acetone (30 ml), and dried under suction for 1 h to give acetyl-d_3_-L-carnitine hydrochloride (6.8 g, 90%) as a white solid.

^1^H NMR (400 MHz, D_2_O) δ 5.70 to 5.61 (1H, m), 3.93 (1H, dd), 3.70 (1H, dd), 3.22 (9H, s), 2.89 (1H, dd), 2.83 (1H, dd).

^13^C NMR (400 MHz, D_2_O) δ 173.1, 172.8, 67.7, 65.4, 54.2, 36.9, 20.0 (m).

### Statistical analyses

All statistical analyses were performed in GraphPad Prism. Statistical specifics are included in figure legends.

## Data availability

All data are contained within the manuscript.

## Supporting information

This article contains supporting information.

## Conflict of interest

The authors declare that they have no conflicts of interest with the contents of this article.

## References

[1] Longo N., Frigeni M., Pasquali M. (2016). Carnitine transport and fatty acid oxidation. Biochim. Biophys. Acta.

[2] Stanley C.A. (2004). Carnitine deficiency disorders in children. Ann. N. Y. Acad. Sci..

[3] Sharma S., Black S.M. (2009). Carnitine homeostasis, mitochondrial function and cardiovascular disease. Drug Discov. Today Dis. Mech..

[4] Hoppel C. (2003). The role of carnitine in normal and altered fatty acid metabolism. Am. J. Kidney Dis..

[5] Qu Q., Zeng F., Liu X., Wang Q.J., Deng F. (2016). Fatty acid oxidation and carnitine palmitoyltransferase I: emerging therapeutic targets in cancer. Cell Death Dis..

[6] McGarry J.D., Brown N.F. (1997). The mitochondrial carnitine palmitoyltransferase system - from concept to molecular analysis. Eur. J. Biochem..

[7] Izzo L., Trefely S., Demetriadou C., Drummond J., Mizukami T., Kuprasertkul N. (2022). The carnitine shuttle links mitochondrial metabolism to histone acetylation and lipogenesis. Cell Biol..

[8] Rasmussen J., Thomsen J.A., Olesen J.H., Lund T.M., Mohr M., Clementsen J. (2015). Carnitine levels in skeletal muscle, blood, and urine in patients with primary carnitine deficiency during intermission of L-carnitine supplementation. JIMD Rep..

[9] Rebouche C.J. (2004). Kinetics, pharmacokinetics, and regulation of l-carnitine and acetyl-l-carnitine metabolism. Ann. N. Y. Acad. Sci..

[10] Parnetti L., Gaiti A., Mecocci P., Cadini D., Senin U. (1992). Pharmacokinetics of IV and oral acetyl-L-carnitine in a multiple dose regimen in patients with senile dementia of Alzheimer type. Eur. J. Clin. Pharmacol..

[11] Juraszek B., Nałęcz K.A. (2019). SLC22A5 (OCTN2) carnitine transporter-indispensable for cell metabolism, a Jekyll and Hyde of human cancer. Molecules.

[12] Salomon J.J., Gausterer J.C., Selo M.A., Hosoya K., Huwer H., Schneider-Daum N. (2019). OCTN2-mediated acetyl-l-carnitine transport in human pulmonary epithelial cells in vitro. Pharmaceutics.

[13] Chen W.W., Freinkman E., Wang T., Birsoy K., Sabatini D.M. (2016). Absolute quantification of matrix metabolites reveals the dynamics of mitochondrial metabolism. Cell.

[14] Trefely S., Huber K., Liu J., Noji M., Stransky S., Singh J. (2022). Quantitative subcellular acyl-CoA analysis reveals distinct nuclear metabolism and isoleucine-dependent histone propionylation. Mol. Cell.

[15] Newman J.C., Verdin E. (2014). Ketone bodies as signaling metabolites. Trends Endocrinol. Metab..

[16] Wang Y., Yu W., Li S., Guo D., He J., Wang Y. (2022). Acetyl-CoA carboxylases and diseases. Front. Oncol..

[17] Altamimi T.R., Thomas P.D., Darwesh A.M., Fillmore N., Mahmoud M.U., Zhang L. (2018). Cytosolic carnitine acetyltransferase as a source of cytosolic acetyl-CoA: a possible mechanism for regulation of cardiac energy metabolism. Biochem. J..

[18] Thul P.J., Åkesson L., Wiking M., Mahdessian D., Geladaki A., Ait Blal H. (2017). A subcellular map of the human proteome. Science.

[19] Houten S.M., Wanders R.J.A., Ranea-Robles P. (2020). Metabolic interactions between peroxisomes and mitochondria with a special focus on acylcarnitine metabolism. Biochim. Biophys. Acta Mol. Basis Dis..

[20] Johnson T.M., Mann W.R., Dragland C.J., Anderson R.C., Nemecek G.M., Bell P.A. (1995). Over-expression and characterization of active recombinant rat liver carnitine palmitoyltransferase II using baculovirus. Biochem. J..

[21] Finocchiaro G., Colombo I., DiDonato S. (1990). Purification, characterization and partial amino acid sequences of carnitine palmitoyl-transferase from human liver. FEBS Lett..

[22] Ceccarelli S.M., Chomienne O., Gubler M., Arduini A. (2011). Carnitine palmitoyltransferase (CPT) modulators: a medicinal chemistry perspective on 35 years of research. J. Med. Chem..

[23] Kim Y., Nam I., Lee D., Bhandari S., Charton L., Kwak S. (2020). Slc25a17 acts as a peroxisomal coenzyme A transporter and regulates multiorgan development in zebrafish. J. Cell. Physiol..

[24] Baker A., Carrier D.J., Schaedler T., Waterham H.R., van Roermund C.W., Theodoulou F.L. (2015). Peroxisomal ABC transporters: functions and mechanism. Biochem. Soc. Trans..

[25] Solberg H.E. (1972). Different carnitine acyltransferases in calf liver. Biochim. Biophys. Acta.

[26] Leonardi R., Zhang Y., Rock C., Jackowski S. (2005). Coenzyme A: back in action. Prog. Lipid Res..

[27] Sanjana N.E., Shalem O., Zhang F. (2014). Improved vectors and genome-wide libraries for CRISPR screening. Nat. Methods.

[28] Doench J.G., Fusi N., Sullender M., Hegde M., Vaimberg E.W., Donovan K.F. (2016). Optimized sgRNA design to maximize activity and minimize off-target effects of CRISPR-Cas9. Nat. Biotechnol..

[29] Sanson K.R., Hanna R.E., Hegde M., Donovan K.F., Strand C., Sullender M.E. (2018). Optimized libraries for CRISPR-Cas9 genetic screens with multiple modalities. Nat. Commun..

[30] Basu S.S., Blair I.A. (2012). SILEC: a protocol for generating and using isotopically labeled coenzyme A mass spectrometry standards. Nat. Protoc..

[31] Snyder N.W., Basu S.S., Worth A.J., Mesaros C., Blair I.A. (2015). Metabolism of propionic acid to a novel acyl-coenzyme A thioester by mammalian cell lines and platelets. J. Lipid Res..

[32] Trefely S., Ashwell P., Snyder N.W. (2016). FluxFix: automatic isotopologue normalization for metabolic tracer analysis. BMC Bioinformatics.

[33] Ichihara K., Fukubayashi Y. (2010). Preparation of fatty acid methyl esters for gas-liquid chromatography. J. Lipid Res..

