# Supplementary material for: Carnitine octanoyltransferase is important for the assimilation of exogenous acetyl-L-carnitine into acetyl-CoA in mammalian cells
Source: J Biol Chem. 2022 Dec 30;299(2):102848. doi: 10.1016/j.jbc.2022.102848 (PMC9898754; doi:10.1016/j.jbc.2022.102848)
Supplement: Supporting Figures S1–S4 and Table S1 [file mmc1.docx]

**Supporting information for:**

Carnitine octanoyltransferase is important for the assimilation of exogenous acetyl-L-carnitine into acetyl-CoA in mammalian cells

Jake Hsu, Nina Fatuzzo, Nielson Weng, Wojciech Michno, Wentao Dong, Maryline Kienle, Yuqin Dai, Anca Pasca, Monther Abu-Remaileh, Natalie Rasgon, Benedetta Bigio, Carla Nasca, Chaitan Khosla^*^

*Address correspondence to [khosla@stanford.edu](mailto:khosla@stanford.edu)

**Supporting Figures**

**Figure S-1**. Representative mass spectra of Acetyl CoA. **(A)** prepared from an authentic standard; **(B)** prepared from wild-type U87MG cells; **(C)** prepared from U87MG treated with ^13^C_2_ LAC; **(D)** prepared from U87MG treated with *d*_3_-LAC.


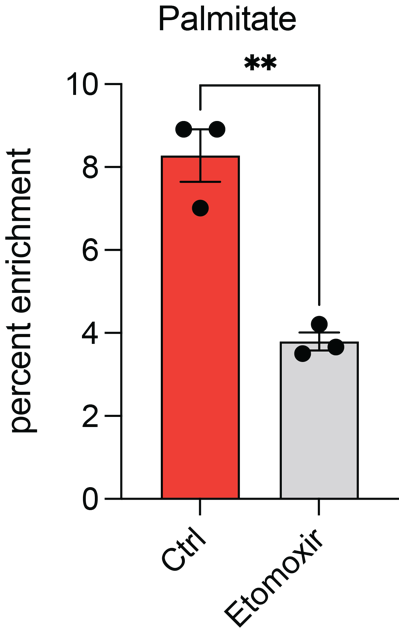


**Figure S-2.** Fractional enrichment of M+2 palmitic acid of cells cultured with 1mM ^13^C_2_ LAC and 10μM etomoxir. Each point represents a biological replicate and error bars represent standard error of the mean. *p≤0.05; **p≤0.01; ***p≤0.001; ****≤p0.0001

**Figure S-3:** Michaelis-Menten analysis of acetyl-, propionyl-, butyryl-, hexanoyl-, and octanoyl-CoA utilization by **(A)** CROT (50 nM) and **(B)** CRAT (10 nM). Each point represents the mean and error bars indicate standard deviation.


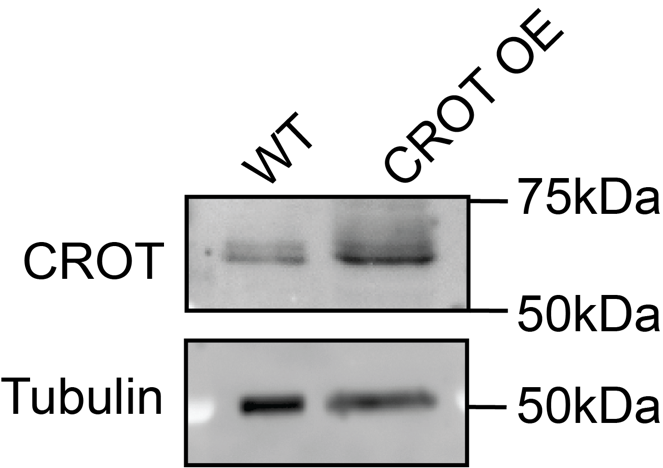


**Figure S-4.** Immunoblot comparing CROT expression in C2C12 myoblasts following transfection with CROT cDNA.

| sgRNA oligos |  |
| --- | --- |
| CRAT | CACCG GCAGCGTCTTGTCGAACCAG |
| CROT | CACCG GATTGCAGCATTAACTAGTG |
| CPT2 | CACCG GATAGGTACATATCAAACCA |

**Table S-1.** gRNA sequences used for CRISPR knockouts.
